# Lysophosphatidylcholine promotes intercellular adhesion molecule-1 and vascular cell adhesion molecule-1 expression in human umbilical vein endothelial cells via an orphan G protein receptor 2-mediated signaling pathway

**DOI:** 10.1080/21655979.2021.1956671

**Published:** 2021-08-04

**Authors:** Qian Zhang, Wei Zhang, Jing Liu, Haisen Yang, Yuxia Hu, Mengdi Zhang, Tuya Bai, Fuhou Chang

**Affiliations:** a The Center for New Drug Safety Evaluation and Research, Inner Mongolia Medical University, Hohhot, China; b The Center for New Drug Screening Engineering and Research of Inner Mongolia Autonomous Region, Inner Mongolia Medical University, Hohhot, China; c College of Pharmacy, Inner Mongolia Medical University, Hohhot, China; d First Clinical Medical College, Inner Mongolia Medical University, Hohhot, China

**Keywords:** LPC, G2A, ICAM-1, VCAM-1, HUVECs, Atherosclerosis, oxLDL, Signaling Pathway

## Abstract

The oxLDL-based bioactive lipid lysophosphatidylcholine (LPC) is a key regulator of physiological processes including endothelial cell adhesion marker expression. This study explored the relationship between LPC and the human umbilical vein endothelial cell expression of intercellular adhesion molecule-1 (ICAM-1) and vascular cell adhesion molecule-1 (VCAM-1) with a particular focus on the regulation of the LPC-G2A-ICAM-1/VCAM-1 pathway in this context. We explored the LPC-inducible role of orphan G protein receptor 2 (G2A) in associated regulatory processes by using human kidney epithelial (HEK293) cells that had been transfected with pET-G2A, human umbilical vein endothelial cells (HUVECs) in which an shRNA was used to knock down G2A, and western blotting and qPCR assays that were used to confirm changes in gene expression. For *in vivo* studies, a rabbit model of atherosclerosis was established, with serum biochemistry and histological staining approaches being used to assess pathological outcomes in these animals. The treatment of both HEK293 cells and HUVECs with LPC promoted ICAM-1 and VCAM-1 upregulation, while incubation at a pH of 6.8 suppressed such LPC-induced adhesion marker expression. Knocking down G2A by shRNA and inhibiting NF-κB activity yielded opposite outcomes. The application of a Gi protein inhibitor had no impact on LPC-induced ICAM-1/VCAM-1 expression. Atherosclerotic model exhibited high circulating LDL and LPC levels as well as high aortic wall ICAM-1/VCAM-1 expression. Overall, these results suggested that the LPC-G2A-ICAM-1/VCAM-1 pathway may contribute to the atherogenic activity of oxLDL, with NF-κB antagonists representing potentially viable therapeutic tools for the treatment of cardiovascular disease.

## GRAPHICAL ABSTRACT

**Figure:**
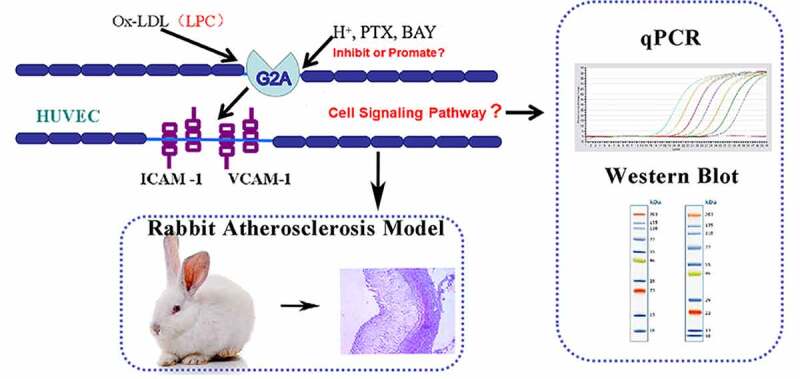

## Introduction

Atherosclerosis is a disease wherein the walls of major blood vessels thicken as a consequence of lipid accumulation, inflammatory cell infiltration, cell death, and thrombus formation, potentially driving myocardial infarction, myocardial ischemia, and mortality. Atherosclerosis is currently the leading cause of death throughout the world [1–4]. While elevated overall cholesterol levels have long been known to be associated with the risk of atherosclerotic disease, there is also growing evidence that low-density lipoprotein (LDL) oxidation is closely related to this pathogenic condition [5–7]. While atherosclerosis and related diseases can exhibit a diverse array of clinical and pathological manifestations, inflammation is a hallmark of all forms of these diseases, and a number of bioactive lipid mediators have been linked to these inflammatory processes [8–10].

Lysophosphatidylcholine (LPC) is a lipid mediator derived from phospholipase A2 (PLA2)-mediated phosphatidylcholine cleavage [11]. It is the main component of oxidized low-density lipoprotein (oxLDL). LPC can bind to Toll-like receptors (TLRs) and G protein-coupled receptors (GPCRs), inducing oxidative stress, lymphocyte and macrophage migration, pro-inflammatory cytokine production, and apoptotic cell death, all of which can exacerbate inflammation and drive related pathological processes [12–18]. By binding to GPCRs in the aorta and other tissue sites, LPC can induce the internalization of these receptors, thereby activating downstream intracellular mitogen-activated protein kinase (MAPK)/extracellular signal-regulated kinase (MEK/ERK) pathways and inducing the chemotactic movement of these cells [19]. By regulating smooth muscle cells (SMCs), endothelial cells (ECs), monocytes, macrophages, and T cells, LPC is thought to shape diverse disease – and inflammation-related processes [20]. For example, LPC can promote adhesion molecule and chemoattractant expression in ECs, drive vascular SMCs proliferation and migration, suppress the migration of ECs following injury, and contribute to abnormal vascular tone [21].

Three key adhesion molecules expressed on EC surfaces, including intercellular adhesion molecule-1 (ICAM-1), E-selectin endothelial-leukocyte adhesion molecule-1 (ELAM-1), and vascular cell adhesion molecule-1 (VCAM-1), have been shown to influence the ability of monocytes and other leukocytes to adhere to endothelial surfaces [22–25]. Low LPC doses can promote selective ICAM-1 and VCAM-1 upregulation in human and rabbit ECs *in vitro*, suggesting it has the potential to influence leukocyte recruitment [26,27]. Indeed, *in vivo* analyses of the mesenteric microvasculature in rats have shown that LPC can promote leukocyte rolling and adherence through the upregulation of ICAM-1 and P-selectin via a mechanism potentially associated with nitric oxide (NO) production given that inhibiting endothelial NO release can induce P-selectin expression [28–30].

**Figure 1.**
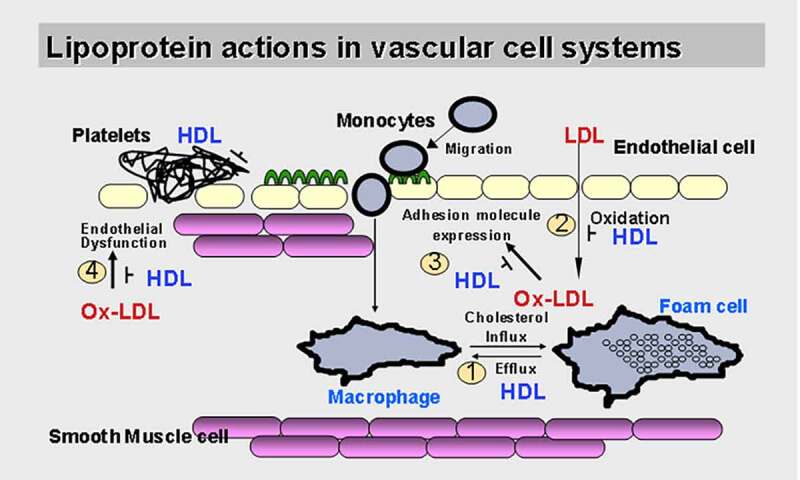
Lipoproteins and atherosclerosis in the vascular system. Low-density lipoprotein, especially oxidized Low-density lipoprotein induces adhesion molecules expression in endothelial cells, but the mechanism of its action is unclear

When ECs are activated by LPC, ICAM-1 and VCAM-1 are significantly upregulated [27]. After entering the inner membrane, monocytes subsequently acquire macrophage characteristics [31]. These biological processes ultimately produce foam cells, which are a hallmark of arterial lesions that secrete proinflammatory cytokines as well as reactive oxygen species (ROS) which amplify the local inflammatory response within the lesion [32] (Figure 1). As mentioned above, both the signaling transduction pathways and the biological effects of LPC have been explored *in vitro* in studies of ECs. Further research, however, is needed to determine whether the biological functions of LPC are related to receptor-mediated or receptor-independent effects mediated by different protein types. Through these specific pathways, LPC can trigger signal transduction cascades involved in the initiation and development of atherosclerosis.

G2A has been shown to regulate proliferation, immune function, oncogenesis, and other important biological pathways, and it shares substantial homology with three other GPCRs including G protein-coupled receptor 4 (GPR4), ovarian cancer G protein-coupled receptor 1 (OGR1/GPR68), and T cell death-associated gene 8 (TDAG8) [33,34]. Both LPC and sphingosylphosphorylcholine (SPC) have been identified as ligands for G2A and GPR4, while SPC is an OGR1 ligand [35], and a glycosphingolipid psychosine has been identified as a TDAG8 ligand [36]. Both OGR1 and GPR4 are proton-sensing GPCRs [37]. Given the vital role of G2A in the pathogenesis of inflammation and immune related diseases, and our previous studies that G2A regulates the LPC-induced expression of ICAM-1 and VCAM-1, we hypothesized that G2A may also be involved in the development of atherosclerosis. To test this, we explored the proton-sensing activity of G2A in this work, with a pH of 6.8 being sufficient to suppress LPC-induced, G2A-mediated ICAM-1 and VCAM-1 expression in human umbilical vein endothelial cells. This suggested that G2A also functions as a proton-sensing GPCR, while LPC is an antagonist for this receptor that controls such proton-dependent G2A activation. To sum up briefly, this study was designed to explore how the LPC/G2A axis regulates the expression of ICAM-1 and VCAM-1 in HUVECs and HEK293 cells in which G2A had been knocked down or overexpressed, with the initial aim of investigating the function of G2A in the pathogenesis of atherosclerosis.

## Materials and methods

### Chemicals and materials

Sixteen New Zealand white rabbits were purchased from Suzhou Huqiao Biotechnology Co., Ltd. (production license number: SCXK (Su) 2015–0002, Suzhou, China). HUVECs and HEK293 cells were purchased from ATCC (VA, USA). DMEM and fetal bovine serum (FBS) were purchased from GIBCO (NY, USA). CCK-8 kits were obtained from Meilun Biotechnology Co., Ltd. (Dalian, China). PTX was purchased from Sigma (MO, USA). pET-G2A, LPC, BAY 11–7085, Anti-ICAM-1, and Anti-VCAM-1 were gifts from Professor Fumikazu Okajima (Gunma University, Maebashi, Japan).

## Cell culture and CCK-8 assay

Both HUVECs and HEK293 cells were cultured in DMEM containing 10% FBS at 37 °C in 5% CO_2_ incubators [38]. For viability assays, cells were plated in 96-well plates for 24 h (5 × 10^3^cells/well) during which they were treated with a range of LPC concentrations (0–100 µmol/L). Media containing 10 µL of CCK-8 reagent was then added (100 µL/well), followed by an additional 3 h incubation [38]. A Multiskan MK3 microplate reader (Thermo Fisher Scientific, USA) was then used to assess absorbance in each well at 450 nm, with viability being established as follows: Cell viability (%) = OD_sample_/OD_control_ ×100%.

## Reverse transcription and real-time quantitative PCR

Trizol (Invitrogen, USA) was used to extract total RNA from appropriately treated HUVECs based upon provided directions, after which M-MLV Reverse Transcriptase (Invitrogen, USA) and oligo(dT) primers (Takara, Japan) were used to prepare cDNA by combining 3 μL of RNA with 1 μL of oligo(dT) primers, 1 μL of 10 mmol/L dNTPs (Promega, USA) and 7 μL of nuclease-free water for 5 min at 65 °C. Tubes were then chilled on ice, after which 4 μL of 5× first-strand buffer, 2 μL DTT, 1 μL of RNAase-out, and 1 μL of M-MLV reverse transcriptase were added, followed by an additional 50 min incubation at 37 °C and a 15 min incubation at 70 °C. GAPDH served as a normalization control for qPCR reactions. Primers were prepared with the Primerexpress software and were synthesized by Invitrogen (Table 1). All qPCR reactions contained 2 μL of PCR buffer, 0.4 μL of each primer (10 μmol/L), 0.4 μL of dNTPs (10 mmol/L), 0.5 μL of cDNA, 0.2 μL of SYBR Green and Taq polymerase (Promega, USA), and nuclease-free water to a final volume of 20 μL. Thermocycler settings were: 94 °C for 5 min; 34 cycles of 94 °C for 1 min, 52 °C for 30 s, and 72 °C for 30 s; 72 °C for 5 min. Melting curve analyses were then performed to ensure the specificity of amplification products. Samples were assessed in triplicate, and the 2^[Ct(GAPDH)-Ct(target gene)]^ approach was used to assess relative gene expression [39].

**Table 1.** Quantitative PCR primers

Gene name	Genebank number	Primer sequence
G2A	NM_013345.2	Forward: 5ʹ-CCTGGTTCTCCTCGTCAAAGC-3’
		Reverse: 5ʹ-TGAGCCTGGTGACGTCTGTCTT-3’
TDAG8	NM_003608.3	Forward: 5ʹ-GTGCAAGGGGAGTGCTTTTCT-3’
		Reverse: 5ʹ-CTTCCCACAACATGACAGCATT-3ʹ
GPR4	NM_005282.2	Forward: 5ʹ-GCTTTCACCAGCCTCAACTGTG-3’
		Reverse: 5ʹ-TGTTCCTCTTGGAGGTGAGTGG −3’
OGR1	NM_ 001177676.1	Forward: 5ʹ-CCACTTCTCCCTCCTGCTCAC −3’
		Reverse: 5ʹ-CGAGTTAGGGGTCTGGAAGGC −3’
GAPDH	NM_002046.3	Forward: 5ʹ-TCAAGTGGGGCGATGCTGGC-3’
		Reverse:5ʹ-TGGGGGCATCAGCAGAGGGG-3’

## G2A overexpression

Lipofectamine 2000 (Invitrogen, USA) was used to transfect HEK293 cells with the pET-G2A or pET-K (empty vector) vectors based upon provided directions [40]. Clones that had been stably transfected were selected using geneticin (1 mg/mL), and G2A expression levels therein were confirmed via qPCR.

## G2A knockdown

HUVECs were grown until 50% confluent, at which time they were transfected with 3.75 μg of G2A shRNA duplexes (5′-CAACGUGUCCUUCGAAGAGtt-3′, 5′-CUCUUCGAAGGACACGUUGtt-3′) or scrambled shRNA duplexes (5′-UUCUCCGAACGUGUCACGUtt-3′, 5′-ACGUGACACGUUCGGAGAAtt-3′) (GenePharma, China) with the TransFast™ Transfection Reagent (Promega, USA) [40]. At 48 h post-transfection, shRNA-mediated knockdown was confirmed via qPCR.

## Cell treatment

HEK293 cells transfected with or without pET-G2A were pretreated with 10 μmol/L LPC, pH 6.8 or pH 6.8 + 10 μmol/L LPC for 24 h at 37 °C in a 5% CO_2_ environment. For analyses of the relationship between G2A signaling and ICAM-1/VCAM-1 expression, HUVECs (1 × 10^5^cells/well) were treated at a pH of 6.8, or with 10 μmol/L LPC, pH 6.8 + 10 μmol/L LPC, 50 ng/mL pertussis toxin (PTX), or 1 μmol/L BAY 11–7085 (an NF-κB inhibitor) for 24 h prior to downstream analyses.

## Western blotting

Following appropriate experimental treatments, cells were rinsed with chilled PBS and scraped from 6-well plates in WIP lysis buffer (Beyotime, China). Lysates were then incubated for 30 min on ice, after which debris was removed by spinning samples for 20 min at 14,000 × g. An equal amount of protein (20 µg/well) was then separated via 7.5% SDS-PAGE and transferred to polyvinylidene difluoride (PVDF) membranes (Millipore, MA, USA) with a Trans-Blot SD semidry transfer cell system (BioRad, CA, USA) using appropriate buffers. Blots were then blocked with 5% fat-free milk in TBST for 1 h, after which they were probed overnight with primary rabbit antibodies specific for ICAM-1, VCAM-1, or β-actin (1:1000, Cell Signaling Technology, MA, USA) at 4 °C. Blots were then washed and incubated for 1 h at room temperature with an anti-rabbit secondary antibody (1:2000), after which protein bands were detected with an Electro-Chemi-Luminescence (ECL) Western Blotting Detection Kit (Beyotime, China) [40].

## Rabbit atherosclerosis model establishment

Sixteen New Zealand white rabbits (eight males, eight females) were fed a high cholesterol diet and injected with BSA to induce atherosclerosis [41]. On week 0, 4, 8 and 12 of the study period, blood samples were collected from the ear veins of all animals in untreated tubes, and a Sapphire 600 Biochemical Analyzer was then used to measure biochemical parameters including total cholesterol (TC), highlow-density lipoprotein (LDL), high-density lipoprotein (HDL), and triglyceride (TG) levels. An arteriosclerosis index (AI) was then calculated for each animal as follows: (TC-HDL)/HDL. ICAM-1 and VCAM-1 levels in aortic tissues for atherosclerotic and control rabbits were assessed via Western blotting [40].

## Hematoxylin and Eosin (H&E) staining

On week 12 of the study period, aorta tissue sections were prepared for H&E staining to assess the degree of atherosclerotic disease induction [41]. Briefly, aortic tissues were fixed for 1 h with 4% paraformaldehyde at 4 °C, rinsed for 5 min with PBS, dehydrated with an ethanol gradient, treated with xylene, and paraffinized. Tissue sections (5 μm) were prepared using an RM2235 rotary microtome (Leica, Germany). After H&E staining, the slices were assessed with a DM2000 microscope (Leica, Germany).

## Thin layer chromatography

A 1 mL serum sample was pretreated with methanol, chloroform, 1 M KCl, and concentrated ammonia (v:v:v:v = 20:30:20:1) and centrifuged at 1000 rpm for 10 min. The lower layer (chloroform) solution was divided into 1.5 mL centrifuge tubes and used for thin-layer chromatography (TLC) [41]. Both the control and arteriosclerosis serum samples as well as LPC standards were loaded onto thin-layer chromatography plates. The mobile phase consisted of chloroform, acetic acid, methanol, ethanol, and water (v:v:v:v:v = 50:12:8:4:1). After the thin layer chromatography (TLC) plates had dried, spots were visualized by spraying them with 0.005% rhodamine 6 G, 0.2% triketohydrindene hydrate, and a molybdenum chromogenic solution (6.5 mM Mo, 4 N H_2_SO_4_, and 46 mM MoO_3_).

## Serum LPC measurement

Serum LPC levels were measured using a phosphate quantitative method [41]. Briefly, the colored spots were scraped off of TLC plates and dissolved with 200 μL of chloroform after which they were centrifuged at 1000 rpm for 10 min. The supernatants, as well as different concentrations of potassium dihydrogen phosphate standard solutions, were pretreated with 200 μL of perchloric acid and digested using an electric furnace at 200 °C for ~1 h. After the solutions had cooled, a total of 5 mL of a solution containing 0.37 mM H_2_SO_4_, 0.3% ammonium molybdate, and 1.2% ascorbic acid were added and the absorbance was measured using a Multiskan MK3 microplate reader (Thermo Fisher Scientific, USA), at 450 nm. LPC contents in the control and arteriosclerosis samples were calculated based upon a potassium dihydrogen phosphate standard curve.

## Statistical analysis

All data were given as means and standard deviations (SDs), and were compared through one-way analyses of variance (ANOVAs) [42] with Bonferroni corrections using SPSS20.0. A value of *P* < 0.05 was considered to indicate statistical significance.

## Results

### *LPC reduces* in vitro *cell viability*

**Figure 2.**
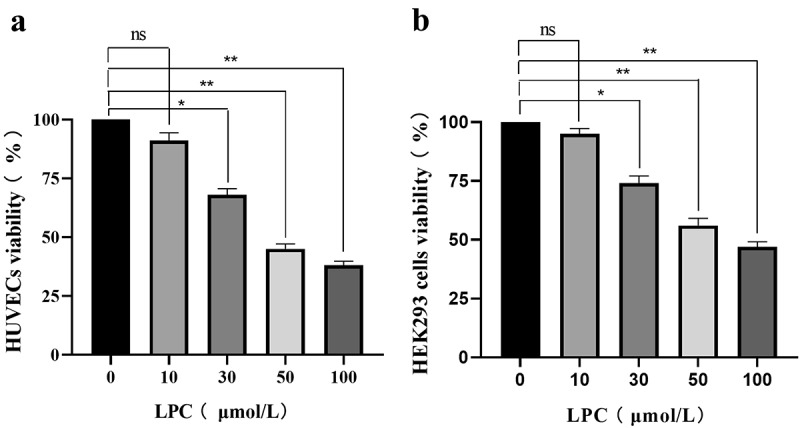
The effects of different LPC concentrations on the viability of HUVECs (a) and HEK293 cells (b). LPC treatment for 24 h decreased cell viability in a dose-dependent manner. n = 3. ^*^P < 0.05, ^**^P < 0.01

We began by examining the cytotoxic impact of LPC by using it to treat HUVECs and HEK293 cells in a CCK-8 assay in order to evaluate the effects on these cells, as such cytotoxicity is crucial to enable subsequent experiments. Following a 24 h treatment, LPC induced dose-dependent toxicity in both cell lines across the tested dose range (0, 10, 30, 50, 100 µmol/L), resulting in approximately 62% and 53% viability at the highest tested dose (Figure 2). Thus, we chose 10 μmol/L LPC for subsequent experiments.

## Analysis of G2A, TDAG8, GPR4, and OGR1 expression in HUVECs

**Figure 3.**
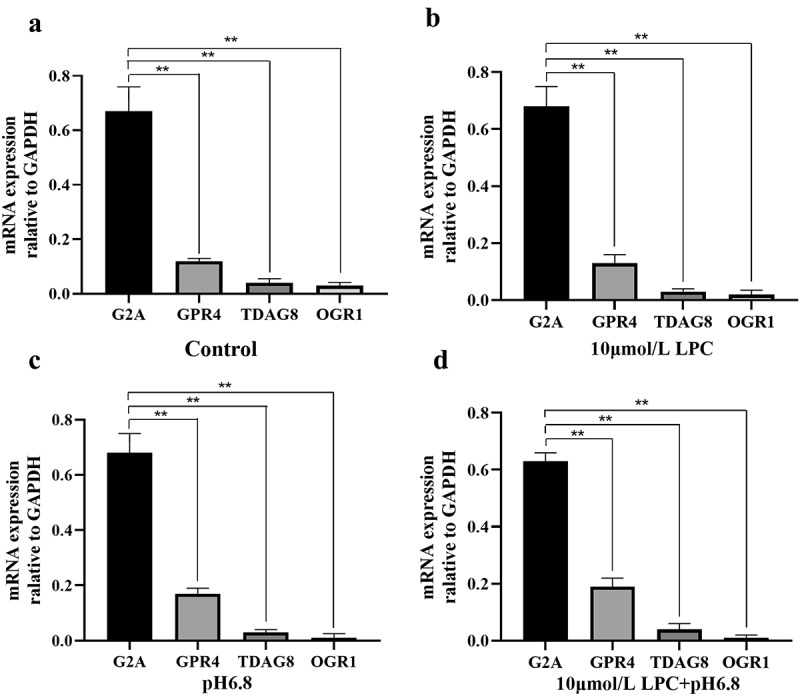
Expression of G2A, GPR4, TDAG8 and OGR1 in HUVECs following control (a), 10 μmol/L LPC (b), pH 6.8 (c), and 10 μmol/L LPC+pH 6.8 treatment. n = 3. ^**^P < 0.01

Given the vital role of G2A in the pathogenesis of inflammation and immune-related diseases, and our previous studies that G2A regulates the LPC-induced expression of ICAM-1 and VCAM-1, we hypothesized that G2A may also be involved in the development of atherosclerosis. To test this, we first examined the expression of G2A, TDAG8, GPR4, and OGR1 in untreated HUVECs. G2A was the most highly expressed of these four receptors. We next employed a qPCR approach to evaluate the effects of LPC treatment and low pH on these four structurally related GPCRs in HUVECs. Cells were treated under the following conditions: pH 6.8, 10 μmol/L LPC, or pH 6.8 + 10 μmol/L LPC. The mRNA levels of these GPCRs were then assessed, revealing that G2A was also the most highly expressed of these four receptors in both control and experimentally treated HUVECs, while OGR1 expression levels was the lowest. LPC treatment and low pH had no apparent effect on the expression format of any of these four receptors (Figure 3). These results confirmed the vital role of G2A in the progression of atherosclerosis and were consistent with our previous hypothesis.

## Stable G2A overexpression in HEK293 cells

**Figure 4.**
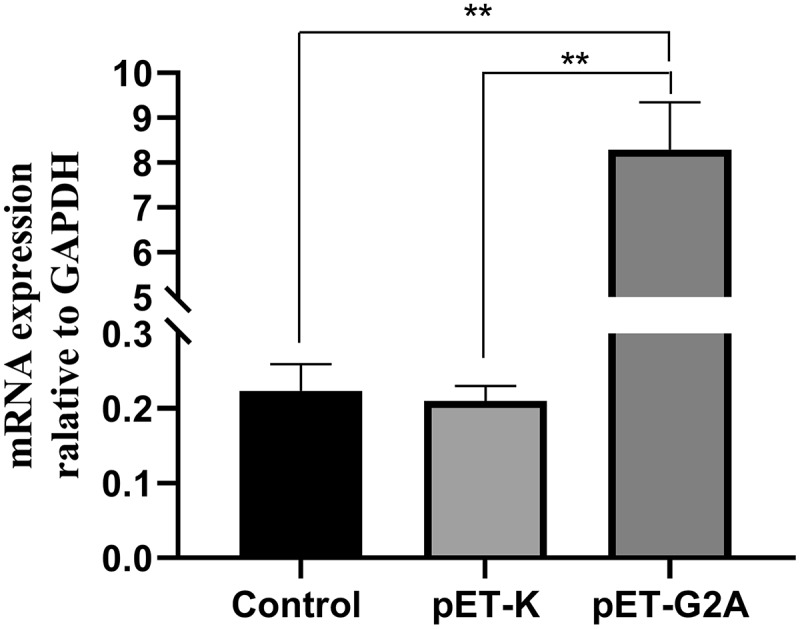
G2A mRNA expression in HEK293 cells following transfection with pET-G2A and pET-K (empty vector), with untreated cells serving as controls. n = 3. ^**^P < 0.01

To evaluate the role of G2A as a regulator of LPC-induced cellular responses, we next transfected HEK293 cells that stably overexpressed a human G2A orthologue (pET-G2A), resulting in a ~ 38-fold increase in G2A expression levels relative to control and pET-K (empty vector) in these cells at 24 h post-transfection (Figure 4).

## G2A knockdown in HUVECs

**Figure 5.**
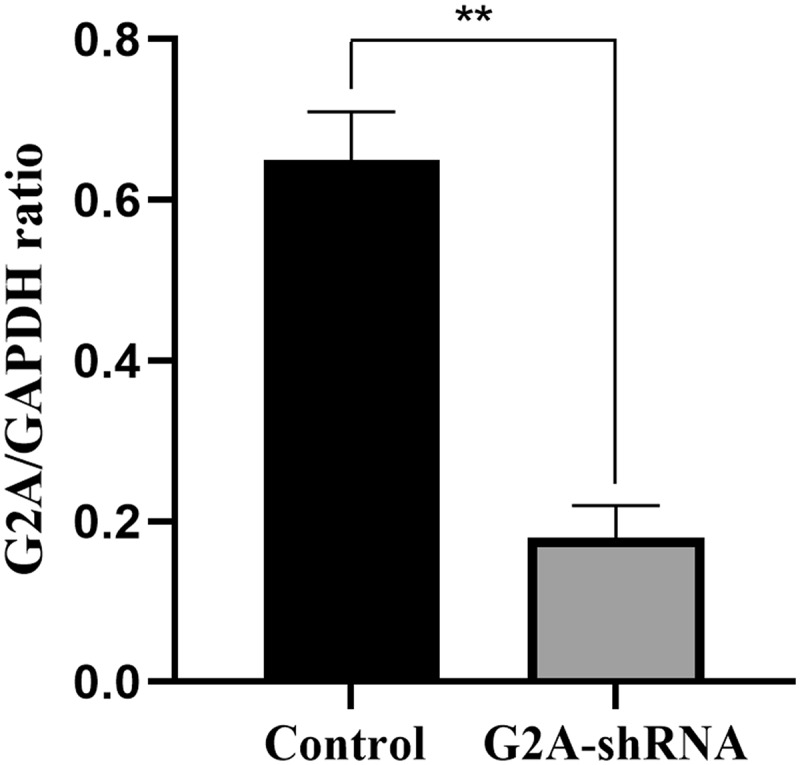
G2A mRNA expression in HUVECs after transfection with an shRNA specific for G2A (G2A-shRNA) or a control scrambled shRNA (Scr-shRNA). n = 3. ^**^P < 0.01

To investigate the role of G2A in LPC‑induced ICAM-1/VCAM-1 expression, G2A expression was silenced using G2A-specific shRNAs in the HUVECs, resulting in a 72% inhibition of G2A expression (Figure 5).

## G2A regulates LPC-induced adhesion marker expression in HEK293 cells

**Figure 6.**
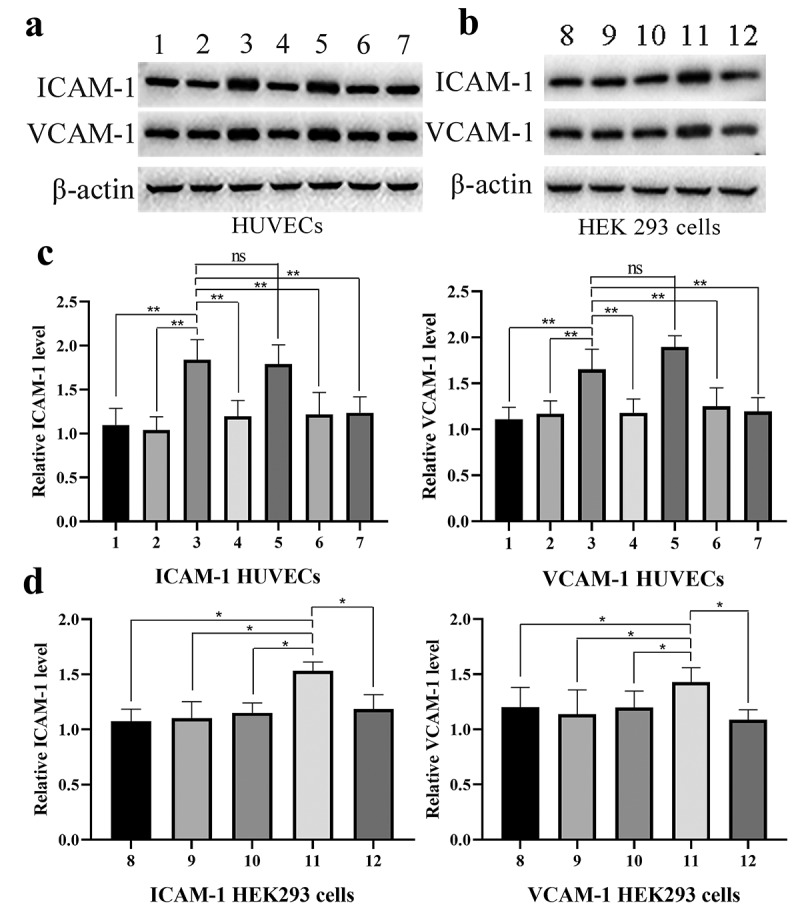
Western blotting was used to measure levels of ICAM-1 and VCAM-1 following control (1), pH 6.8 (2), 10 μmol/L LPC (3), 10 μmol/L LPC+pH 6.8 (4), 10 μmol/L LPC+50 ng/mL PTX (5), 10 μmol/L LPC+1 μmol/L BAY 11–7085 (6), 10 μmol/L LPC+ shG2A (7) treatment in HUVECs (a), and following control (8), pH 6.8 (9), 10 μmol/L LPC (10), 10 μmol/L LPC+pET-G2A (11), 10 μmol/L LPC+pET-G2A +pH 6.8 (12) treatment in HEK293 cells. n = 3. ^*^P < 0.05, ^**^P < 0.01

As HEK293 cells express low G2A levels and exhibit high transfection efficiency, yielding high-quality recombinant proteins, we next assessed the relationship between G2A and the LPC-induced expression of ICAM-1 and VCAM-1 in HEK293 cells transfected with the pET-G2A or pET-K constructs. These cells were treated for 24 h under control, pH 6.8, LPC (10 μmol/L), or pH 6.8 + LPC (10 μmol/L) conditions, after which adhesion marker expression was assessed. While no changes in ICAM-1 or VCAM-1 expression were observed in control cells (Figure 6b and Figure 6d), LPC stimulation significantly, whereas a pH of 6.8 inhibited such LPC-induced ICAM-1 and VCAM-1 expression.

## G2A regulates LPC-induced adhesion marker expression in HUVECs

To examine the relationship between G2A and LPC-induced ICAM-1/VCAM-1 expression in further detail, we analyzed the levels of these adhesion markers via Western blotting following treatment at a pH of 6.8, with LPC (10 μmol/L), with PTX (50 ng/mL), or with BAY 11–7085 (1 μmol/L). This analysis revealed that the levels of these proteins were unchanged in the control and pH 6.8 groups, whereas they were significantly increased following LPC stimulation. Notably, a pH of 6.8 suppressed LPC-induced ICAM-1/VCAM-1 upregulation (Figure 6a and Figure 6c). G2A knockdown in these HUVECs resulted in reductions in the expression of ICAM-1/VCAM-1 following LPC treatment. To examine whether NF-κB influenced the LPC-induced upregulation of these adhesion markers, we treated these cells with BAY 11–7085 for 1 h, and found that such treatment strongly suppressed LPC-induced ICAM-1/VCAM-1 upregulation (Figure 6a and Figure 6c). We further determined that PTX had no impact on this pathway, suggesting that LPC functions in this context via Gi-independent signaling mechanisms. Together with the above results, these data suggest that G2A mediates the LPC-induced upregulation of ICAM-1 and VCAM-1 in endothelial cells.

## LPC-induced ICAM-1/VCAM-1 expression is linked to the incidence of atherosclerosis *in vivo*

**Figure 7.**
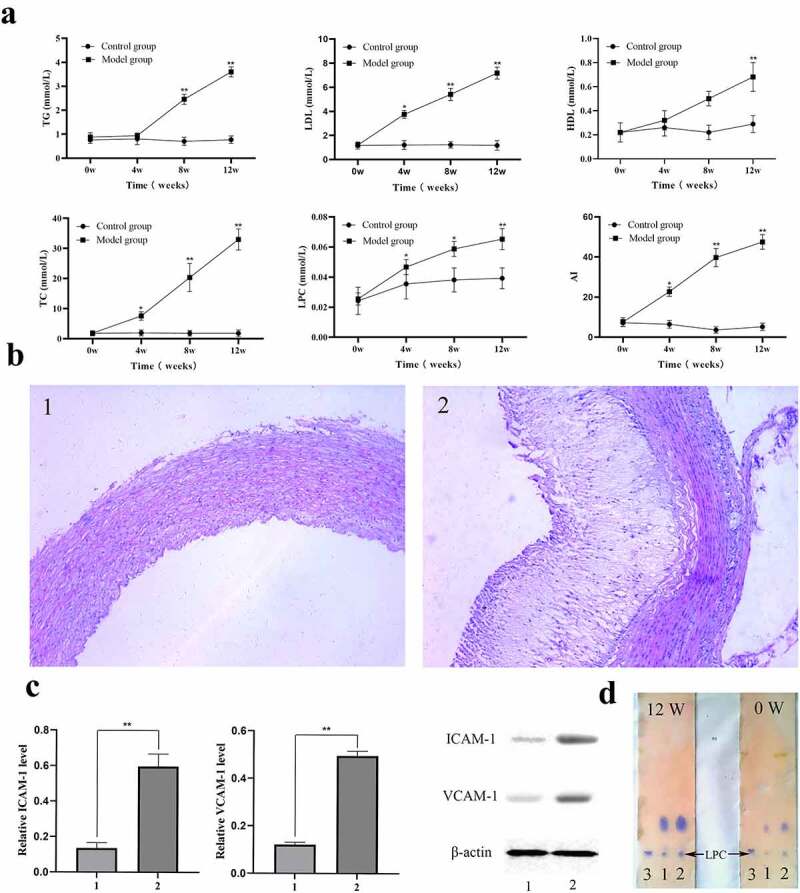
*In vivo* analysis of the link between LPC-induced ICAM-1/VCAM-1 expression and atherosclerosis. Blood samples were collected from control and model groups at 0, 4, 8, 12-week time points, and the levels of TC, LDL, HDL, TG, and atherogenic indexes (AI) were measured with a biochemical analyzer (a). Blood samples were collected from control (1) and model groups (2), and were measured via TLC along with an LPC standard (3), in addition to being measured via a phosphate quantitative method (d). Aortas from control (1) and model group (2) rabbits were subjected to H&E staining (b), and the ICAM-1 and VCAM-1 protein levels from control (1) and model group (2) rabbits were measured via Western blotting (c). n = 6 or n=3. ^*^P < 0.05, ^**^P < 0.01

To verify the effect of the LPC-G2A-ICAM-1/VCAM-1 pathway on atherosclerosis, we next established the atherosclerotic rabbit models. TC, LDL, HDL, TG, and LPC levels in model and control animals were then measured and used to calculate an atherogenic index. On week 12 of the study period, the atherogenic index values for model group animals were ~9-fold higher than control animals (Figure 7a). LPC levels in model group animals were significantly higher than in control rabbits as determined via thinlayer chromatography (Figure 7a and Figure 7d). ICAM-1 and VCAM-1 expression were also significantly elevated in aortic tissue samples from model group rabbits relative to healthy controls (Figure 7c). This suggested that the LDL-oxLDL-LPC axis may regulate ICAM-1/VCAM-1 expression in the context of atherosclerosis.

## Histological assessment of atherosclerotic disease induction

Next, we investigated the involvement of LDL-oxLDL-LPC in the formation of atherosclerotic plaques *in vivo* using traditional rabbit arteriosclerosis models. Following blood sample collection, animals underwent gross necropsy. Arteriosclerotic plaques were evident on the inner aortic wall in model group rabbits, whereas these walls remained smooth in control aortic tissue samples. H&E staining of aortic tissue sections was performed to assess the extent of these atherosclerotic lesions, revealing significant neointimal thickening in the model group relative to the control group (Figure 7b).

## Discussion

LPC induces pro-inflammatory effects including the upregulation of adhesion molecules in endothelial cells, the increased release of interleukin-1β (IL-1β), interleukin-6 (IL-6), and tumor necrosis factor-α (TNF-α) from adipocytes, the enhanced secretion of interferon-γ from peripheral blood mononuclear leucocytes, and the increased activation of B cells and macrophages [43–45]. These actions are thought to be mediated through G2A signaling pathways. Orphan GPCRs lacking characterized ligands have recently been shown to bind to LPC, with LPC being reported to bind to both G protein-coupled receptor 132 (G2A/GPR132) and G protein-coupled receptor 4 (GPR4), thereby influencing cellular growth and immune cell activation [46,47]. Monocytes and macrophages express high levels of G2A, and LPC can stimulate T cell and macrophage chemotaxis in a G2A-dependent fashion [48]. G2A may thus be an important mediator of the recruitment of these immune cell types to arterial walls in atherosclerotic contexts [49]. Parks et al. [50] have also previously reported that G2A^−/ –^ LDLR^−/ –^ mice exhibit a reduction in macrophage and T cell accumulation within lesion-prone aortic sites, with increased circulating HDL cholesterol levels following treatment for extended periods of time with a western diet. G2A may thus contribute to *in vivo* atherosclerotic disease progression through mechanisms associated with altered leukocyte chemotaxis, although it may also influence this pathogenic condition by modulating lipoprotein metabolism [50]. In contrast, Bolick et al. [51] demonstrated that a loss of G2A in mice was associated with increased interactions between ECs and monocytes within the aorta, with endothelial G2A expression thus preventing atherosclerosis and associated vascular inflammation. With respect to GPR4, Lum et al. [52] determined that human vascular ECs expressed GPR4 but not G2A at the mRNA level, suggesting that LPC may primarily shape EC atherosclerotic responses in a GPR4-dependent manner. Qiao et al. [53] additionally found that siRNA-mediated GPR4 knockdown was sufficient to partially protect against LPC-induced RhoA activation, stress fiber formation, and associated decreases in transendothelial resistance. This led the authors to propose that endogenous GPR4 is a key mediator of inflammatory EC responses to LPC. Huang et al. [54] confirmed that LPC was sufficient to enhance RhoA activation and monocyte transmigration while decreasing the expression of GPR4 was sufficient to block these responses to LPC. In contrast, low pH did not affect this pathway [54]. Whether LPC interacts directly with G2A or GPR4 is still controversial, their activation seems to nonetheless be linked to the atherosclerotic effects of LPC in a range of pathophysiological states. Further study of these and other specific LPC receptors is thus critical to fully understand the mechanisms whereby it can shape the onset and progression of atherosclerosis.

Through a series of *in vitro* assays, we determined that LPC was able to promote ICAM-1/VCAM-1 upregulation. In prior studies, LPC has been shown to regulate cell growth and immune cell responses by interacting with the proton-sensitive G2A and GPR4 GPCRs. Murakami et al. [55] suggested that in this context, LPC serves as an antagonistic compound that controls proton-dependent G2A activation. G2A has not been previously studied in HUVECs, and we herein found that of four homologous GPCRs, it was expressed at the highest level relative to levels of OGR1, GPR4, and TDAG8 in these cells. Meanwhile, G2A expression format was not impacted by LPC exposure or acidic conditions (pH 6.8). We therefore hypothesized that G2A serves as an LPC receptor and thereby influences the biological responses of HUVECs to LPC exposure.

We then explored the impact of LPC on HUVECs in which G2A had been knocked down, revealing a marked decrease in LPC-induced ICAM-1/VCAM-1 upregulation. We additionally found that the overexpression of G2A using the pET-G2A vector in HEK293 cells resulted in increased ICAM-1/VCAM-1 expression following LPC exposure. Through the overexpression and knockout of the G2A gene, we were able to establish that G2A plays an indispensable role in the LPC-induced expression of ICAM-1 and VCAM-1. For both HUVECs and HEK293 cells, LPC treatment thus altered the proton-sensitive activation of G2A. Local environmental acidification and LPC production may interact to promote pathogenicity in certain disease contexts. Indeed, prior data support the hypothesis that G2A is a pH-sensitive receptor that plays such a role in physiological contexts. Further studies evaluating animal models of G2A deficiency will clarify these issues and help establish the physiological role of these proteins.

The LPC-mediated activation of G2A and subsequent signal transduction govern the ability of this molecule to influence cellular physiology, controlling a range of vital processes such as proliferation and migration [56]. The use of PTX as an inhibitor of Gi proteins had no impact on the ability of LPC to induce the expression of these adhesion molecules, suggesting this pathway to be Gi-independent. NF-κB is a key inflammatory transcription factor that plays a role in the pathogenesis of atherosclerosis. To evaluate whether inhibiting NF-κB might alter ICAM-1/VCAM-1 expression in our model system, we treated HUVECs with a specific NF-κB inhibitor (BAY 11–7085), and observed significant reductions in the levels of these adhesion markers at 24 h following treatment. As such, BAY 11–7085-mediated NF-kB inhibition may thus be an effective approach to inhibiting chronic inflammation and thereby preventing atherosclerotic disease progression.

Overall, our data suggest that oxLDL may drive atherosclerotic plaque formation at least in part via the LPC-mediated upregulation of ICAM-1 and VCAM-1. We observed significant increases in the levels of LDL, LPC and these adhesion markers in atherosclerotic model rabbits, and we determined that serum LDL and LPC levels were significantly altered in these animals relative to controls. Levels of LDL particles, which are composed of triglycerides, phospholipids, and cholesterol, are directly associated with the severity of atherosclerotic disease. While passive LDL retention was originally thought to drive the progression of atherosclerosis, more recent research has instead shown that LDL can undergo an array of oxidative modifications within the vasculature including esterification and lipid peroxidation, yielding oxLDL [57]. In addition to being potentially antigenic, oxLDL can directly promote inflammation and the activation of innate and adaptive immune responses [57]. For example, in circulation, oxLDL can enhance the ability of monocytes to bind to ECs by promoting the expression of ICAM-1/VCAM-1. These leukocytes are then able to develop into foam cells containing high levels of lipids. Overall, we herein found that HUVECs express high levels of G2A, which is thus a promising biomarker of atherosclerosis that can mediate the LPC-induced upregulation of ICAM-1/VCAM-1 via Gi-independent mechanisms. These findings highlight the potential biological role of LPC and provide new cellular and molecular insights into the mechanisms whereby LPC may modulate cell signaling pathways. Future studies regarding the role of vascular signaling mechanisms and related diseases should continue to focus on the physiological and therapeutic methods that inhibit the LPC signal transduction cascade.

Although key aspects of the process of atherosclerotic plaque development and progression such as local inflammation, LDL oxidation, macrophage activation, and necrotic core formation have already been studied elucidated, many molecular mechanisms affecting this process have yet to be clarified. For example, phospholipid biochemistry is complex, animal models of human atherosclerotic disease are limited in many respects, and establishing causal relationships between particular lipids within vascular walls and the incidence of clinical outcomes is challenging, complicating research efforts in this field. Additionally, the optimal level of LPC in the plasma has not been established, and the mechanisms underlying the harmful effects of LPC are not well understood. Recent advances and novel targets, particularly in the field of RNA interference-based therapies or specific antagonists, hold great promise. However, it should be noted that the modulation of a particular gene is not as clearly associated with a complex polygenic disease as it is in the case of monogenic diseases. A deeper understanding of the molecular mechanisms underlying atherosclerosis, further progress in the field of vector development, and the demonstration of treatment efficacy in relevant animal models will be required before gene therapy-based atherosclerosis treatment can become a clinical reality.

## Conclusions

In summary, our data suggest that the LPC-G2A-ICAM-1/VCAM-1 pathway may mediate the atherogenic activity. Together with our previous results, these findings suggest that targeting G2A in combination with NF-κB antagonists or inhibitors may be an effective approach to suppressing atherosclerosis and associated cardiovascular disease. Future studies aim at determining the physiological significance of G2A-mediated effects in the context of inflammation and chronic inflammatory disease, and establishing the lipid specificity of such effects, should therefore be conducted using animal models with cell-specific G2A deficiencies or transgenic G2A expression together with the genetic modification of key enzymes, such as PLA2, that are responsible for LPC generation. These studies should also incorporate accurate approaches to quantifying levels of LPC and other lipids that regulate G2A activity, including lyso-PS and oxidized free fatty acids. Such approaches will be required to determine whether targeting G2A activity or its interaction with LPC in specific immunoregulatory cell types represents a viable approach to beneficially modulating inflammatory and immunological processes in the context of chronic inflammatory and autoimmune disease.

## Data Availability

All data generated or analysed during this study are included in this published article

## Abbreviations

oxLDL oxidized Low-density lipoprotein; ICAM-1 intercellular adhesion molecule-1 ; VCAM-1 vascular cell adhesion molecule-1; LPC lysophosphatidylcholine; G2A orphan G protein receptor 2; HEK293 human kidney epithelial; HUVECs human umbilical vein endothelial cells; qPCR quantitative polymerase chain reaction; NF-κB nuclear factor kappa-B; LDL low-density lipoprotein; PLA2 phospholipase A2; TLRs Toll-like receptors; GPCRs G protein-coupled receptors; ECs endothelial cells; SMCs smooth muscle cells; MAPK mitogen-activated protein kinase; MEK/ERK extracellular signal-regulated kinase; ELAM-1 E-selectin endothelial-leukocyte adhesion molecule-1; NO nitric oxide; ROS reactive oxygen species; GPR4 G protein-coupled receptor 4; LDLR low-density lipoprotein receptor; HDL high-density lipoprotein; mRNA messenger RNA; OGR1/GPR68 ovarian cancer G protein-coupled receptor 1; TDAG8 T cell death-associated gene 8; SPC sphingosylphosphorylcholine; ATCC American-type culture collection; FBS fetal bovine serum; DMEM dulbecco’s modified eagle medium; DTT dithiothreitol; GAPDH glyceraldehyde-3-phosphate dehydrogenase; PTX pertussis toxin; shRNA short hairpin RNA; PBS phosphate buffer saline; SDS-PAGE sodium dodecyl sulfate polyacrylamide gel electrophoresis; PVDF polyvinylidene fluoride; ECL Electro-Chemi-Luminescence; TC total cholesterol; TG triglyceride; AI arteriosclerosis index; H&E Hematoxylin and Eosin; TCL thin layer chromatography; ANOVA one-way analysis of variance; SRs scavenger receptors; IL-1β IL-interleukin-1β; IL-6 interleukin-6; TNF-α tumor necrosis factor-α.

## Highlights

- G2A knockdown led to a reduction in ICAM-1/VCAM-1 expression following LPC treatment.
- G2A overexpression led to an increase in ICAM-1/VCAM-1 expression following LPC treatment.
- A pH of 6.8 inhibited LPC-induced ICAM-1/VCAM-1 expression.
- LPC directly targeted G2A and drove ICAM-1/VCAM-1 upregulation.
